# Supplementary material for: Enhanced in vivo blood brain barrier transcytosis of macromolecular cargo using an engineered pH-sensitive mouse transferrin receptor binding nanobody
Source: Fluids Barriers CNS. 2023 Aug 24;20:64. doi: 10.1186/s12987-023-00462-z (PMC10463325; doi:10.1186/s12987-023-00462-z)
Supplement: Supplementary file 1 — Additional file 1. Supplemental information, including supplemental methods, supplemental results and figures, supplemental tables, supplemental discussion, and supplemental references. [file 12987_2023_462_MOESM1_ESM.pdf]

SUPPLEMENTAL INFORMATION

"Enhanced *in Vivo* Blood Brain Barrier Transcytosis of Macromolecular Cargo Using an Engineered pH-sensitive Mouse Transferrin Receptor Binding Nanobody"

Thomas J. Esparza, Shiran Su, Caroline M. Francescutti, Elvira Rodionova, Joong Hee Kim, David L. Brody

Supplemental Methods

Quantification of nanobody biotinylation

The degree of biotin incorporation was determined using the Pierce Biotin Quantitation Kit (#28005, Thermo Scientific). The assay measures the displacement of avidin from HABA (4'-hydroxyazobenzene-2-carboxylic acid) with nanobody-conjugated biotin. Briefly, the HABA/avidin complex was reconstituted in ultra-pure water and a 1/10 dilution was prepared in 1xPBS. The absorbance at 500nm was recorded for a baseline measurement. The biotinylated nanobody samples were subsequently diluted in the HABA/avidin reagent, incubated briefly and the absorbances at 500nm were measured. The Δ500nm measurement of the reagent alone and diluted biotin-nanobody were then used to determine the molar equivalents of biotin in the nanobody sample as outlined in the manufacturer's operating protocol.

Quantification of Claudin-5 by ELISA

The concentration of claudin-5 in capillary-depleted brain homogenates and the isolated capillary fractions were measured using a commercially available ELISA kit (#CSB-EL005507MO, Cusabio). Briefly, capillary-depleted homogenates and isolated capillary fractions were prepared and supplemented with 0.5% (v/v) radioimmunoprecipitation assay (RIPA) buffer. Total protein was measured for each sample using a micro-BCA protein assay (#23235, Thermo Scientific). The claudin-5 assay was performed according to the manufacturer's protocol, with the minor modification of supplementing the standard curve and all dilutions with RIPA buffer at 0.5% (v/v) to normalize any effect of RIPA buffer on assay binding. The samples and standard were loaded onto the ELISA plate and incubated overnight at 4°C. The assay was then developed following the manufacturer protocol and the absorbance measured at 450nm. The sample concentrations were normalized to total protein as reported in picograms claudin-5/milligrams total protein.

VE-Cadherin Western blot in mouse brain homogenates

Naïve wild-type mouse brain homogenates were processed to deplete the capillary component as described above. Gel electrophoresis was performed by separating 20-micrograms of total protein per lane of both pre-capillary-depletion and post-capillary-depletion brain homogenates on a 10% Bis-Tris acrylamide gel, including size standards. Following electrophoresis, the gel was transferred to 0.2 µm nitrocellulose blotting membrane using the wet-transfer method in tris-glycine buffer at 100 volts for 2hr in a chilled transfer tank. The transferred membrane was blocked using a 3% (w/v) bovine serum albumin in 1xPBS solution for 1 hour with gentle agitation. The VE-cadherin primary antibody (#ab205336, Abcam) was diluted 1:1000 (v/v) in blocking buffer and exchanged onto the membrane and allowed to incubated overnight at 4°C with gentle agitation. Following three washes with 1xPBS for 10 minutes each, goat anti-rabbit-peroxidase antibody (#ab97051) solution was prepared in the blocking buffer and exchanged onto the membrane for 1 hour at room-temperature with gentle agitation. A final triple wash was performed, and the membrane was developed using Clarity Max Western ECL (#1705062, BioRad) reagent and the chemiluminescence imaged on a BioRad ChemiDoc image station using standard acquisition parameters.

Supplemental Results and Figures

We wished to assess the relative importance of affinity at pH 7.4 vs. pH dependent unbinding under acidic conditions for *in vivo* NT-mediated hypothermia. To do so, we plotted the extent of NT-induced hypothermia after intravenous injection of 25 nmol/kg of each M1-NT construct vs. each parameter (**Suppl. Fig. 1A-B**). We found that there was no significant monotonic relationship between affinity and NT effect (Spearman r=0.01, p=0.96); there were NT constructs with strong NT effects with affinities ranging from 6 to 246 nM, and other NT constructs with similar affinities that did not have strong NT effects. Instead, there was a clearer correlation of NT-induced hypothermia *in vivo* with the fold increase in unbinding at pH 5.5 vs. 7.4 (Spearman r=0.4, p=0.004). However, even this parameter only explained a modest proportion of the variance in NT-induced hypothermia. Notably, several NT constructs with substantially increased unbinding at pH 5.5 such as M1$_{R56H, P96H, Y102H}$ still did not induce hypothermia *in vivo.* In a three-dimensional plot of affinity at pH 7.4 and pH-dependent unbinding vs. NT-induced hypothermia, we observed that the combination of affinity in the range of 6 to 246 nM plus at least 2-fold greater unbinding at pH 5.5 vs pH 7.4 was strongly associated with potent NT-induced hypothermia (**Suppl. Fig. 1C**). There were still outliers; the M1$_{Y98H, Y102H}$ mutant for example mediated substantial NT-induced hypothermia (5°C), with only modestly greater unbinding at pH 5.5 vs pH 7.4 and a low affinity (>5000 nM). Thus, it appears that both optimal affinity and pH dependent unbinding contribute substantially but do not fully explain the NT-induced hypothermia mediated by the M1 histidine mutants. Nonetheless, the M1$_{R56H, P96H, Y102H}$ mutant with strongly pH dependent unbinding and preserved high affinity was highly effective at producing NT-mediated hypothermia *in vivo.*

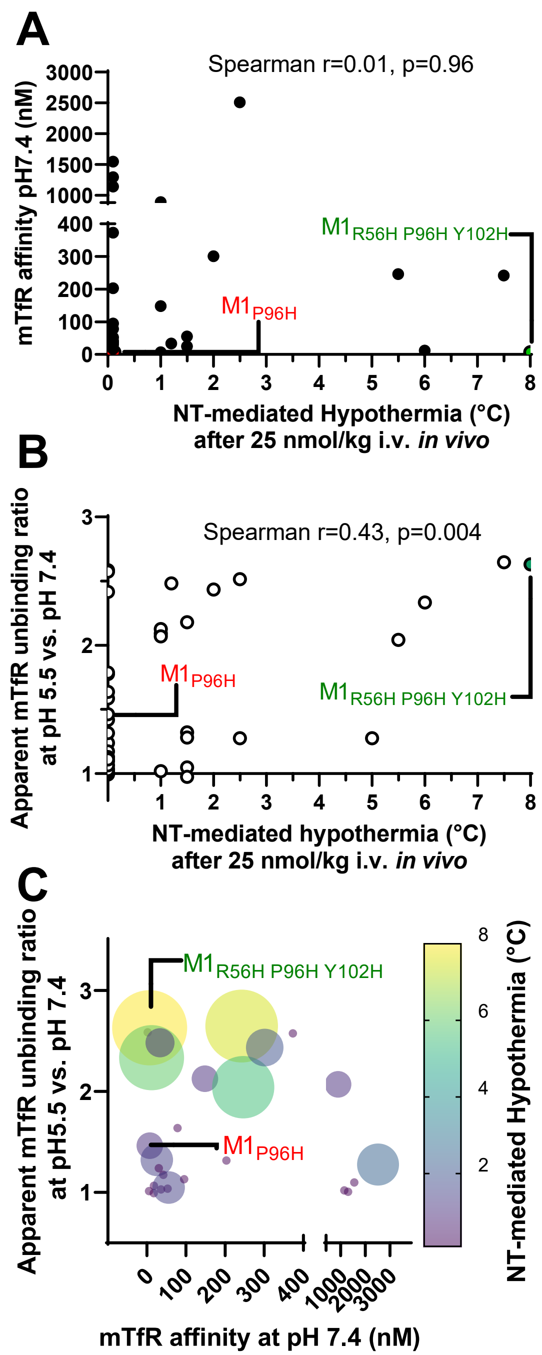

Suppl. Fig.1. *In vitro* TfR binding vs. *in vivo* NT-mediated hypothermia for multiple M1 histidine mutants. **A.** NT-mediated hypothermia *in vivo* vs. mTfR binding measured by ELISA at pH 7.4 for constructs with measurable affinities. **B.** NT-mediated hypothermia *in vivo* vs. the ratio of apparent mTfR unbinding at pH 5.5 vs. unbinding at pH 7.4 as measured by ELISA. **C.** NT-mediated hypothermia vs. both affinity and apparent pH 5.5 vs pH 7.4 unbinding ratio. NT-mediated hypothermia expressed as both bubble size and color. NT-mediated hypothermia was measured in a blinded fashion after intravenous injection of 25 nmol/kg of each construct (n=3 mice per construct).

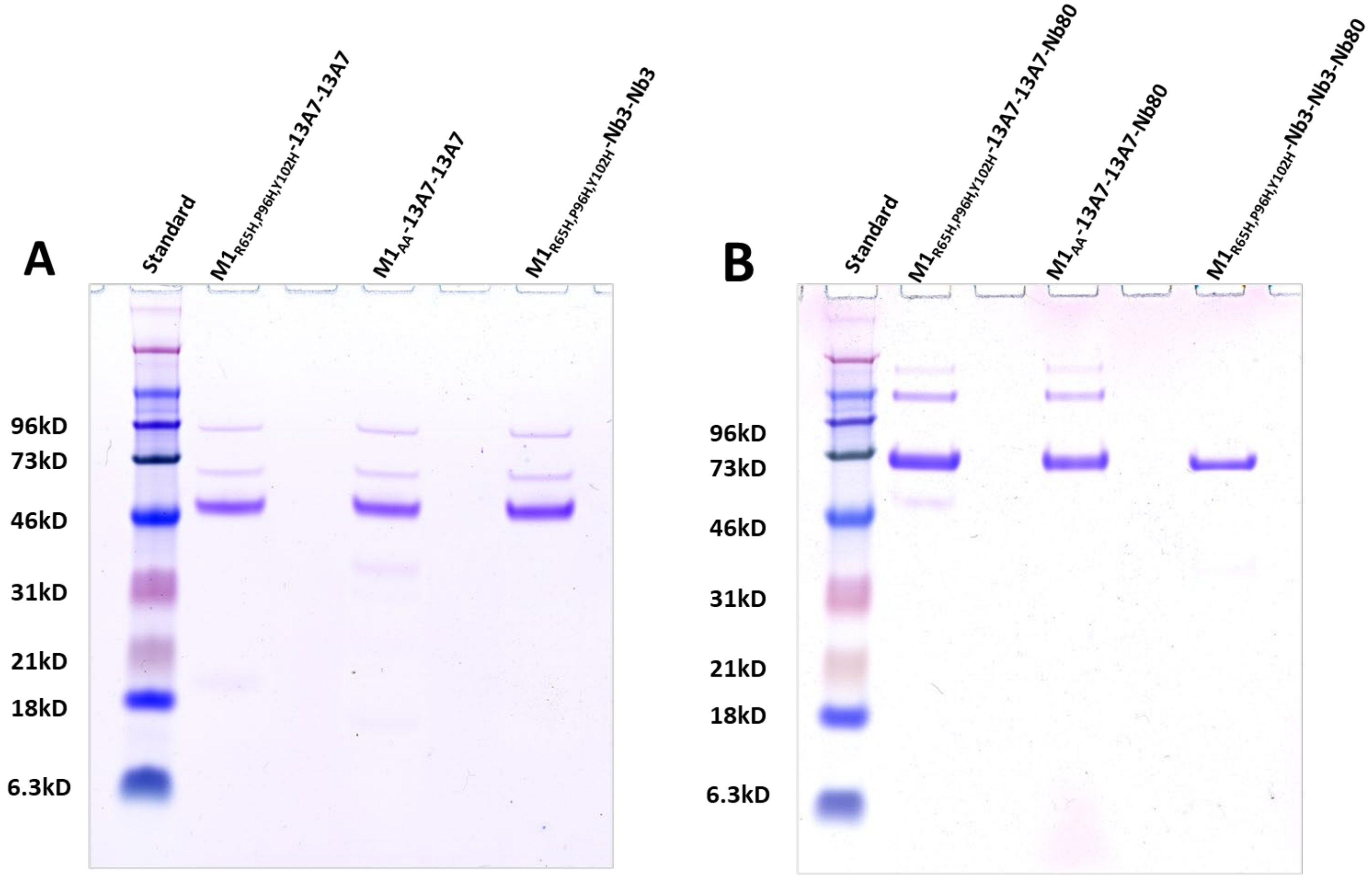

Suppl. Fig. 2. Sodium dodecyl sulfate polyacrylamide electrophoresis (SDS PAGE) assessment of nanobody construct purification. **A** Three nanobody constructs. **B.** Four nanobody constructs.
Following metal-affinity purification and size-exclusion chromatography to purify each construct, SDS-PAGE was performed on 10% Bis-Tris gels and stained with Coomassie blue R-250. Size standard molecular weight indicated in kilodaltons (kD). Band densities were analyzed using ImageJ. The primary band at the expected size was found to constitute >70% of the total for all constructs.

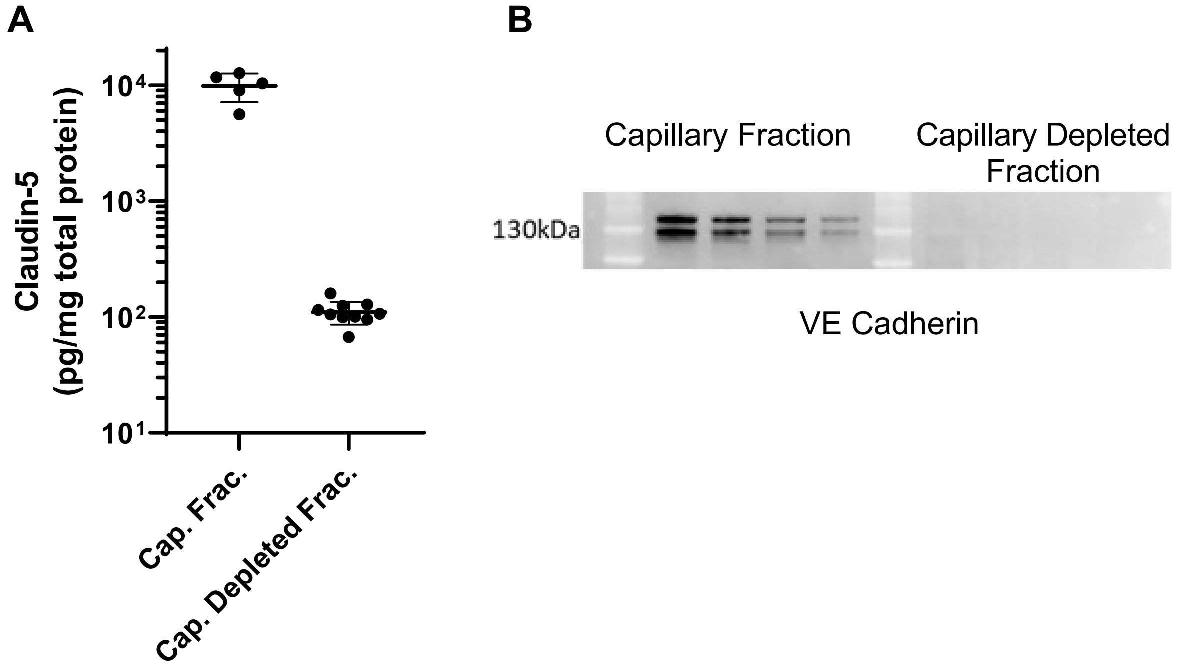

Suppl. Fig. 3. Verification of capillary depletion from brain lysates. **A.** Claudin-5 ELISA of lysates from the capillary containing fractions and capillary depleted fractions of mouse brains after injection of M1-based nanobody constructs. Note log scale of y-axis. Capillary depletion removes ~99% of Claudin-5, a brain capillary endothelial marker. **B.** VE Cadherin western blotting of lysates from the capillary containing fractions and capillary depleted fractions. After capillary depletion, there was no detectible VE Cadherin, another brain capillary endothelial marker.

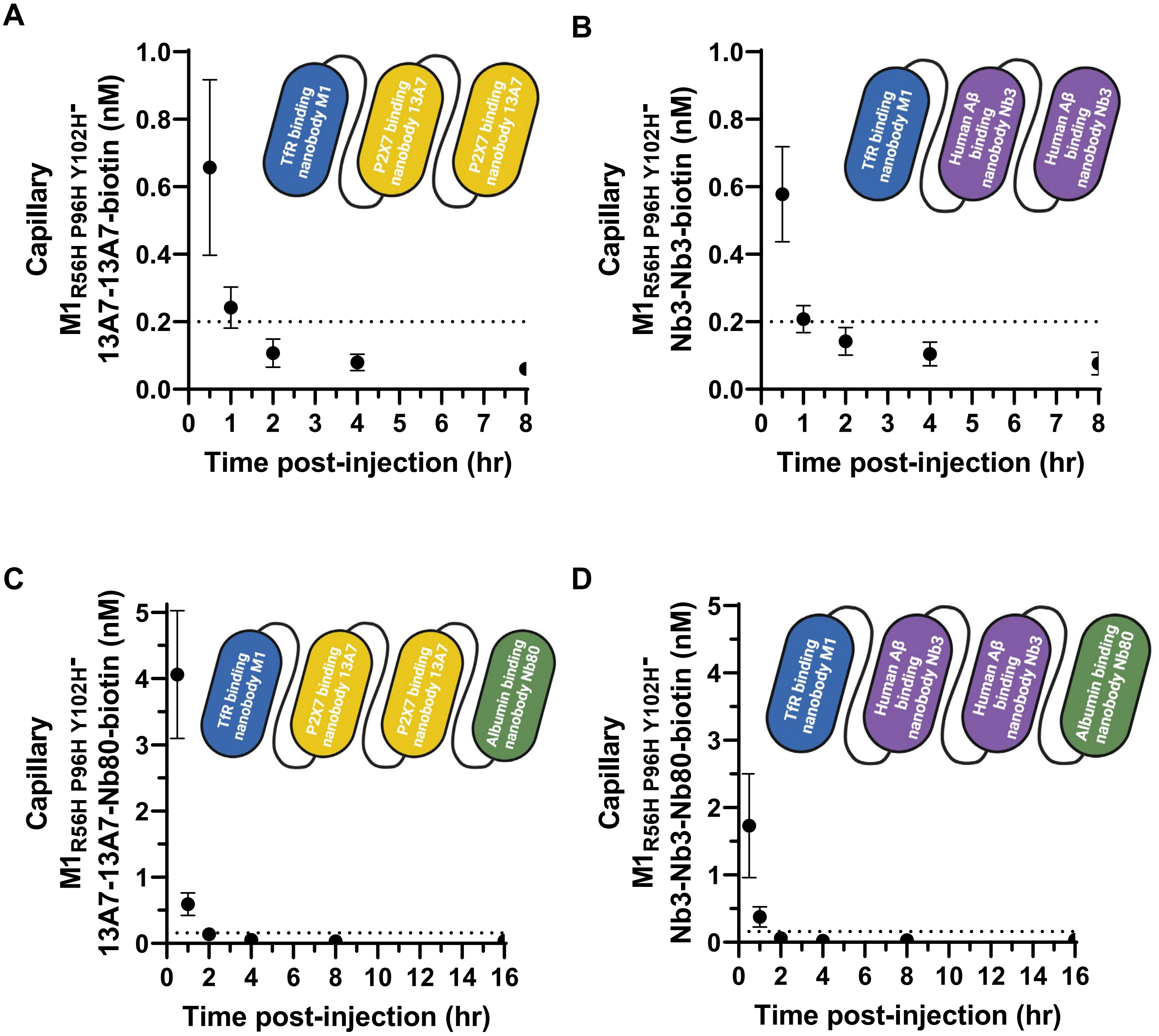

Suppl. Fig. 4. TfR-binding nanobody construct concentrations in capillary containing fractions of brain lysates. **A.** Three nanobody construct with P2X7 receptor target binding. **B.** Three nanobody construct with no brain target binding. **C.** Prolonged blood half-life four nanobody construct with P2X7 receptor target binding. **D.** Prolonged blood half-life four nanobody construct with no brain target binding.

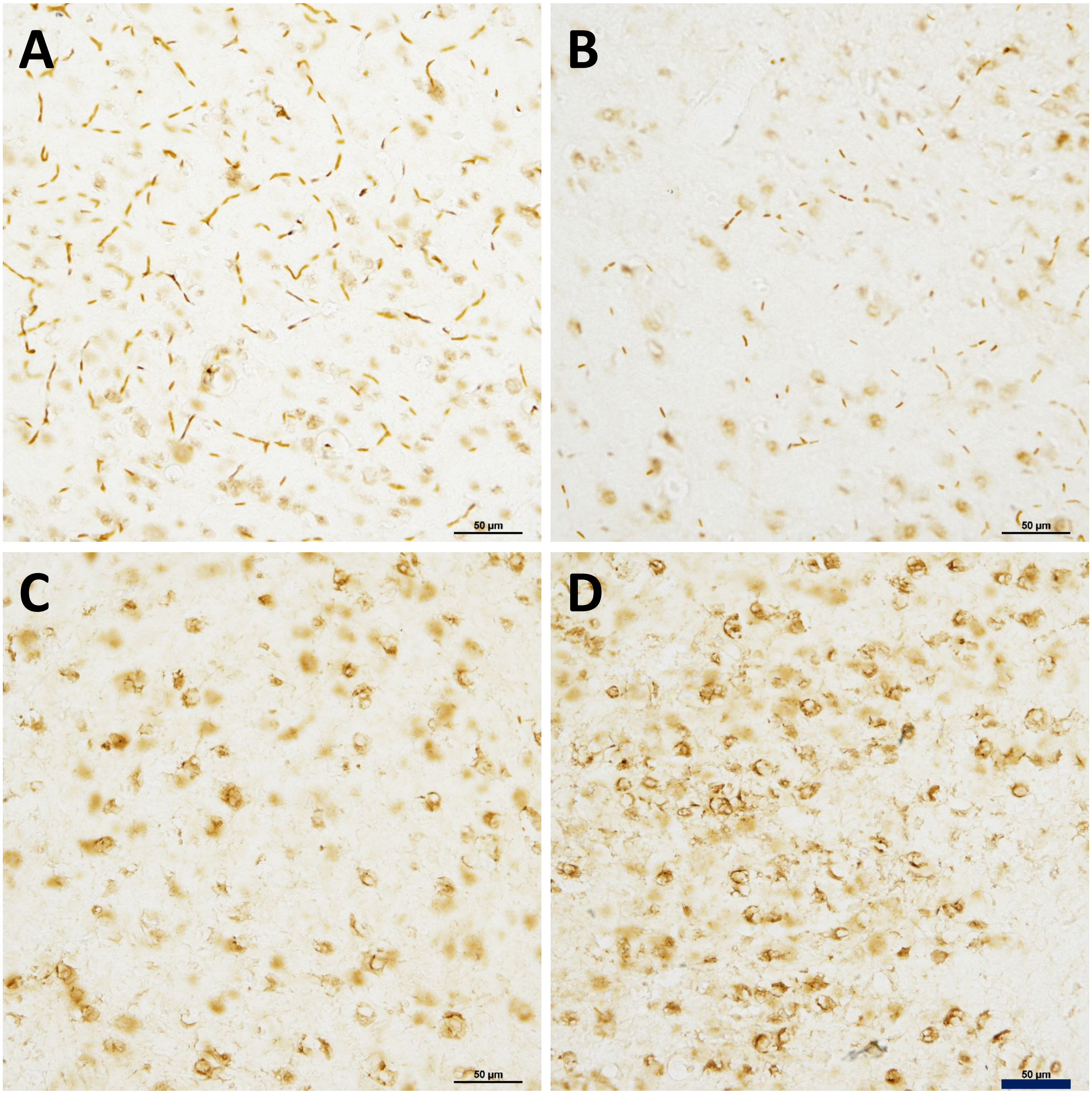

Suppl. Fig. 5. High magnification (40x) images from *in situ* labeling of injected biotinylated $M1_{R56H,\ P96H,\ Y102H}$-13A7-13A7-Nb80 construct in mouse hippocampus. **A, B**. *In situ* labeling of biotinylated $M1_{R56H,\ P96H,\ Y102H}$-13A7-13A7-Nb80 injected iv into wild-type mice at 600 nmol/kg body weight sacrificed 30 min after injection. **C, D**. *In situ* labeling of biotinylated $M1_{R56H,\ P96H,\ Y102H}$-13A7-13A7-Nb80 injected iv into wild-type mice at 600 nmol/kg body weight sacrificed at 4 hrs after injection. (Scale bars = 50 µm)

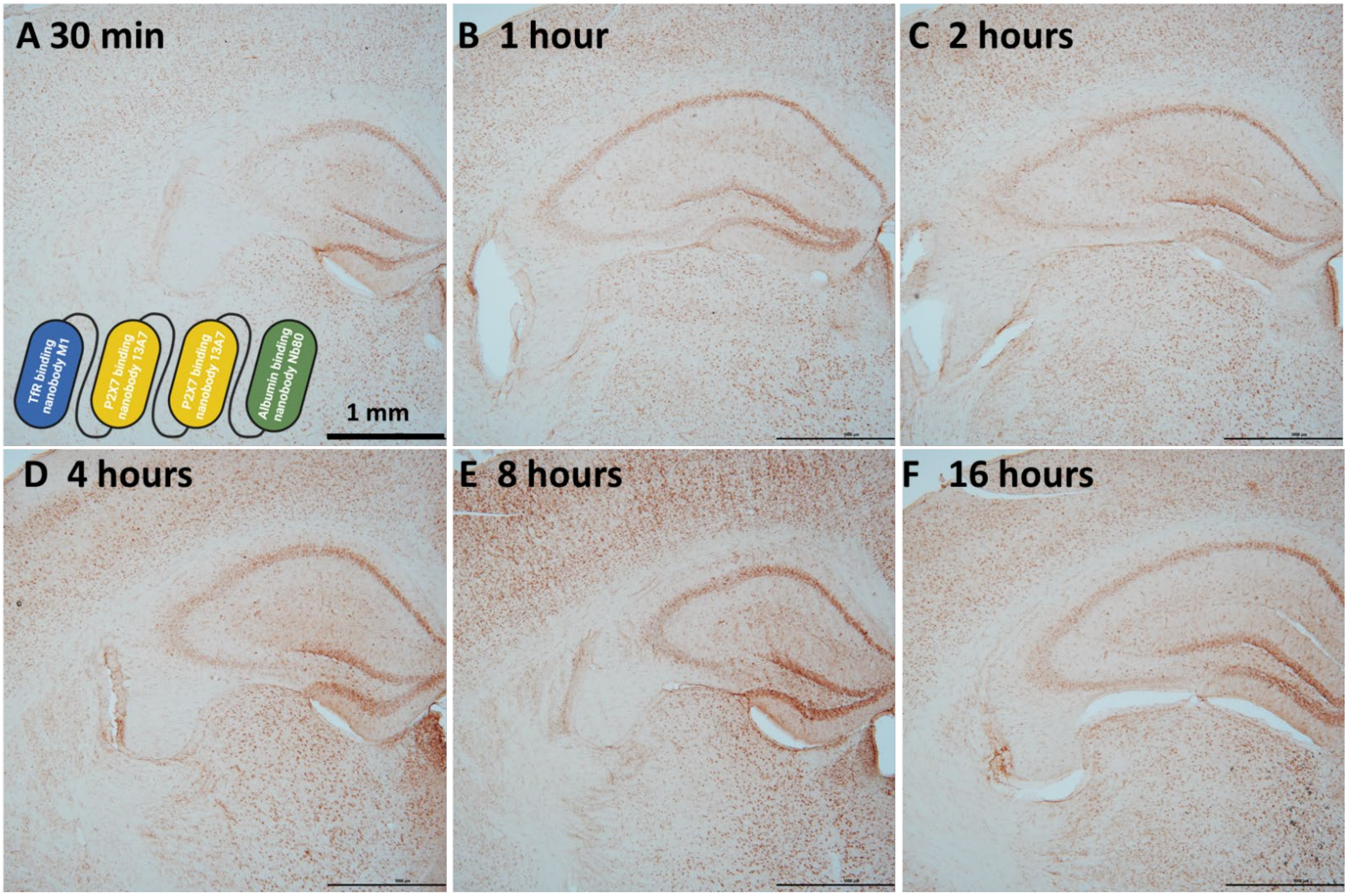

Suppl. Fig. 6. Lower magnification (4x) images from *in situ* labeling of biotinylated $M1_{R56H,\ P96H,\ Y102H}$-13A7-13A7-Nb80 injected iv into wild-type mice at 600 nmol/kg body weight from the same mice as in Fig. 5. Both cortex and hippocampus at 30 min show labelling in structures with morphologies consistent with capillaries. There was widespread cellular labeling at 1, 2, 4, 8 and 16 hrs in hippocampus, cortex, white matter and thalamus.

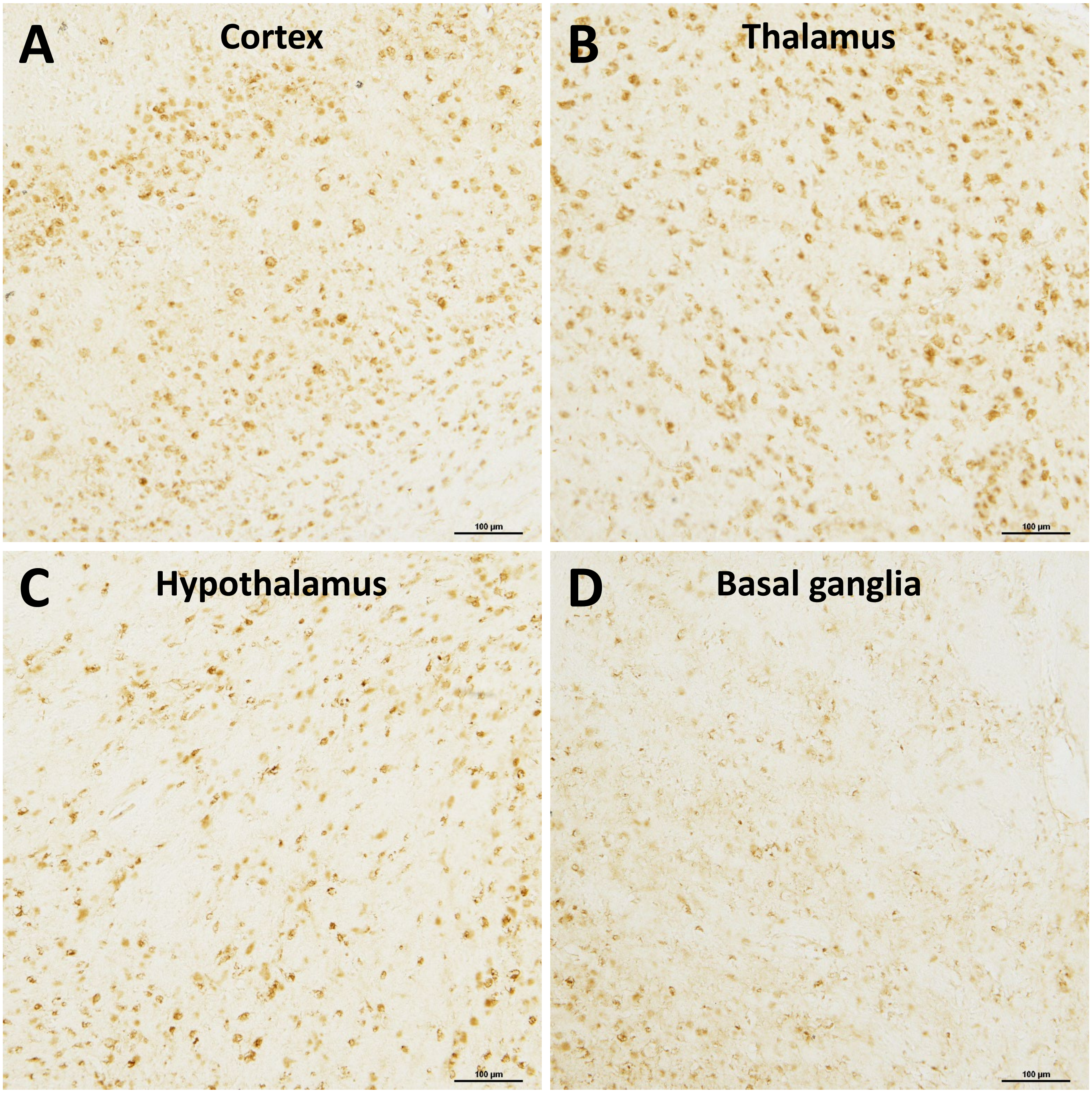

Suppl. Fig. 7. I*n situ* labeling of injected biotinylated $M1_{R56H,\ P96H,\ Y102H}$-13A7-13A7-Nb80 construct in other mouse brain regions. *In situ* labeling of biotinylated $M1_{R56H,\ P96H,\ Y102H}$-13A7-13A7-Nb80 injected iv into wild-type mice at 600 nmol/kg body weight and sacrificed 4 hrs after injection demonstrating cellular staining in (**A**) cortex, (**B**) thalamus, (**C**) hypothalamus, and (**D**) basal ganglia. (20x magnification. Scale bars = 100 µm)

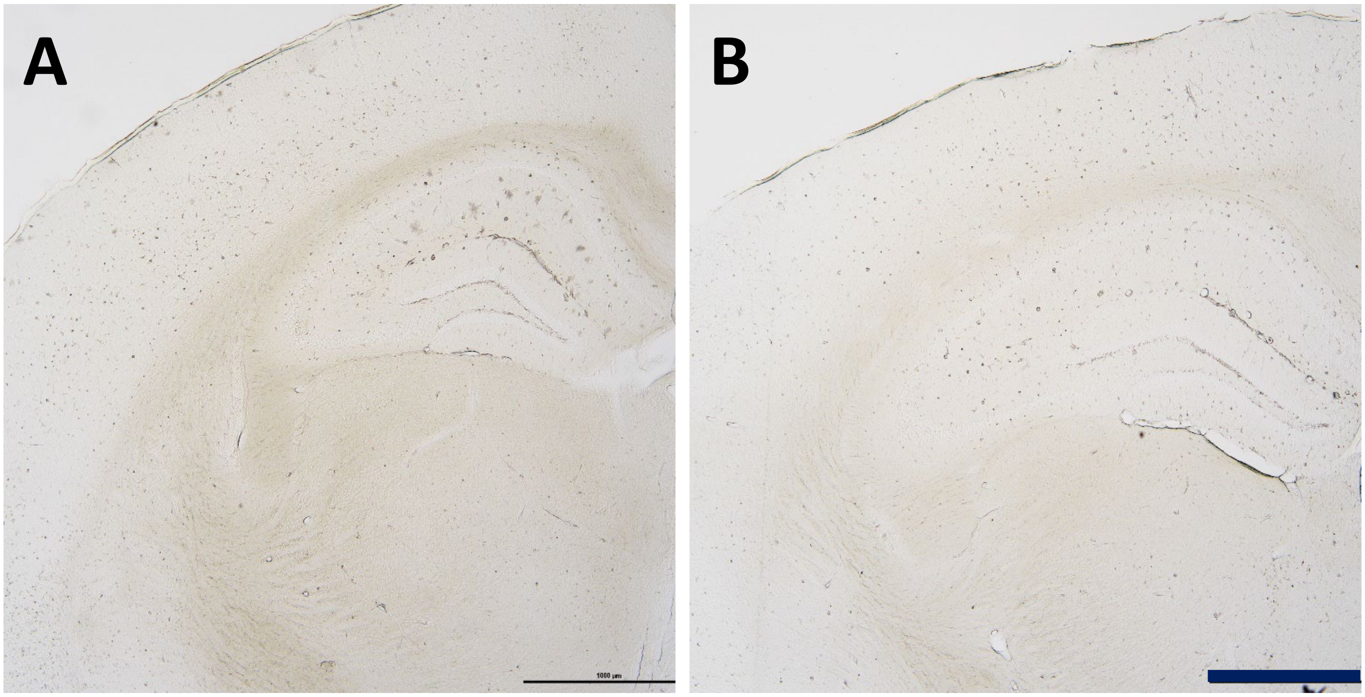

Suppl. Fig. 8. Low magnification (4x) images from *ex vivo* labeling of control nanobodies. **A.** *Ex vivo* staining of naïve WT mouse brain slice with 50 nM biotinylated M1$_{R56H,\ P96H,\ Y102H}$. **B.** *Ex vivo* staining of naïve WT mouse brain slice with 50 nM biotinylated M1$_{R56H,\ P96H,\ Y102H}$-Nb3-Nb3-Nb80. (Scale bars = 1000 µm)

Supplementary Tables:

**Nanobody Construct (time after injection)**	**Dose (nmol / kg)**	**Mouse body weight (g)**	**Injected dose (nmol)**	**Brain sample mass (mg)**	**Homog. volume (mL)**	**Brain Conc. (nM)**	**Brain quantity (nmol)**	**Quantity per gram brain (nmol)**	**% ID/g brain**
$M1_{R56H, P96H, Y102H}$ – 13A7-13A7 (1 hour)	30	20.5±1.8	0.62	223±8	1	2.3	0.0023	0.0103	1.7
$M1_{R56H, P96H, Y102H}$ – 13A7-13A7 (4 hrs)	30	22.0±1.3	0.66	227±6	1	1.6	0.0016	0.0070	1.1
$M1_{R56H, P96H, Y102H}$ – Nb3-Nb3 (30 min)	30	23.2±1.0	0.69	220±7	1	1.46	0.0015	0.0066	0.96
$M1_{R56H, P96H, Y102H}$ – Nb3-Nb3 (4 hrs)	30	19.4±0.8	0.58	217±13	1	0.49	0.0005	0.0022	0.39
$M1_{R56H, P96H, Y102H}$ – 13A7-13A7-Nb80 (4 hrs)	1	21.5±1.3	0.02	221±7	1	0.64	0.0006	0.0029	13.4
$M1_{R56H, P96H, Y102H}$ – 13A7-13A7-Nb80 (4 hrs)	3	21.5±1.0	0.06	213±5	1	1.21	0.0012	0.0057	8.81
$M1_{R56H, P96H, Y102H}$ – 13A7-13A7-Nb80 (4 hrs)	10	21.6±1.9	0.22	214±8	1	2.30	0.0023	0.0107	4.98
$M1_{R56H, P96H, Y102H}$ – 13A7-13A7-Nb80 (4 hrs)	30	21.4±1.0	0.64	208±2	1	4.69	0.0047	0.0225	3.51
$M1_{R56H, P96H, Y102H}$ – 13A7-13A7-Nb80 (4 hrs)	100	21.4±1.6	2.14	211±5	1	4.40	0.0044	0.0209	0.97
$M1_{R56H, P96H, Y102H}$ – 13A7-13A7-Nb80 (4 hrs)	300	21.1±0.4	6.33	210±5	1	4.04	0.0040	0.0192	0.30
$M1_{R56H, P96H, Y102H}$ – 13A7-13A7-Nb80 (4 hrs)	1000	22.0±0.5	22.0	211±1	1	3.12	0.0031	0.0148	0.07
$M1_{R56H, P96H, Y102H}$ – 13A7-13A7-Nb80 (4 hrs)	3000	21.0±0.6	63.0	215±2	1	3.28	0.0033	0.0153	0.02
$M1_{R56H, P96H, Y102H}$ – Nb3-Nb3-Nb80 (4 hrs)	30	18.8±0.3	0.56	221±4	1	0.94	0.0009	0.0043	0.75

Suppl. Table 1. Percent injected dose per gram of brain (%ID/g) calculations (n=3 per group).

Reference	BBB crossing approach	Macromolecule cargo	Dose injected	Time after injection	% Inj. Dose / gram Brain
Friden et al., 1991 [3]	Rat TfR IgG OX-26	Methotrexate via Fc linked carbohydrate in rats	~30 nmol/kg	24 hours	0.27%
Boado et al., 2010	Mouse TfR IgG	Anti-Aβ scFv	~ 5 nmol/kg	1 hour	3.5% total (not capillary depleted)
Yu et al., 2011 [30]	Mouse TfR IgG low affinity	BACE1 bispecific antibody in WT mice	~133 nmol/kg	12-48 hours	0.6-0.8%
Zuchero et al., 2016 [13]	CD98hc IgG	Anti-BACE1 in WT mice	Tracer (not specified)	1-48 hours	1-6%
Sehlin et al., 2016 [65]	Mouse TfR IgG 8D3	mAb158 vs Aβ protofibrils in TgArcSwe mice	~3 nmol/kg	4-72 hours	0.15 - 0.25%
Boado & Pardridge 2017 [8]	Insulin R IgG	Iduronidase in rhesus	0.1 mg/kg	2 hours	1.1% ID/100 g brain
Hultqvist et al., 2017 [66]	Mouse TfR scFv 8D3 x2	mAb158 in TgArcSwe mice	0.25 nmol/kg<br>50 nmol/kg	72 hours	2.2%<br>0.33% -0.75%
Sonoda et al., 2018 [6]	Human TfR IgG	hIDS in cyno monkeys	20 nmol/kg	24 hours	~0.4%
Wicher et al., 2019 WO 2019/ 246288A1	CD98hc VNAR	Human Fc	25 nmol/kg	3-4 hours	1.3% total (not capillary depleted)
Kariolis et al., 2020 [32]	Human TfR binding IgG Fc	BACE 1 IgG in human TfR mice	300 nmol/kg	24 hours	~3.4%
Logan et al., 2021 [67]	Human TfR binding IgG Fc	Progranulin in GRN-/- mice	40 nmol/kg<br>400 nmol/kg	24 hours	0.25%<br>0.05%
Shin et al., 2022 [12]	Anti-IGF1R scFv	Alpha-synuclein IgG in mice	340 nmol/kg	24 hours	0.4%
Van Lengerich et al., 2023 [68]	Human TfR binding IgG Fc	Activating mTREM2 IgG 4D9 in human TfR mice	66 nmol/kg	1 day	1.1-1.5%
Gehrlein et al., 2023 [69]	Mouse TfR binding IgG Fab	Recombinant gluco-cerebrosidase in WT and transgenic mice	~15 nmol/kg	24 h	~3%
Esparza et al., (this paper)	Mouse TfR nanobody $M1_{R56H,\ P96H,\ Y102H}$	13A7-13A7 nanobodies in WT mice	30 nmol/kg	1 hour	1.7%
Esparza et al., (this paper)	Mouse TfR nanobody $M1_{R56H,\ P96H,\ Y102H}$	13A7-13A7-Nb80 nanobodies in WT mice	1 nmol/kg<br>3 nmol/kg<br>30 nmol/kg	4 hours	13.4%<br>8.8%<br>3.5%

Suppl. Table 2. Percent injected dose/gram of brain for the P2X7 receptor binding $M1_{R56H,\ P96H,\ Y102H}$-13A7-13A7 three nanobody construct and $M1_{R56H,\ P96H,\ Y102H}$-13A7-13A7-Nb80 four nanobody construct compared with previously published BBB shuttles assessed using similar capillary depletion brain homogenate methods. When detailed methods were not specified, we assumed that mouse body weight was 20 grams and that 200 mg brain samples were lysed in 1 ml for these calculations. WT: Wild-type.

Supplemental Discussion

The nanobody constructs developed here have relatively fast kinetics and may be potentially useful for short-acting therapeutics and diagnostic applications. However, for applications where a long duration of action is favorable such as treatment of chronic brain diseases, solutions based on receptor mediated transcytosis using conventional immunoglobulins may be preferred [6] [8] [12] [13] [30] [31] [32]. It may be possible to further prolong the duration of action of nanobody-based constructs by making hybrid nanobody-IgG fusions, conjugation to polyethylene glycol, or other approaches, but these may negate many of the advantages of nanobody-based constructs noted elsewhere. Similarly, nanobody-based constructs to not induce IgG Fc-mediated immune responses; this may be an advantage for some applications and a disadvantage for others. Furthermore, for applications where high doses of macromolecules must be delivered to relatively localized targets, focal ultrasound mediated blood brain barrier opening [70] [71] [72] may be preferred over global delivery via brain shuttle systems.

The concentrations of the nanobody constructs in the extracellular space of the brain have not been measured directly. The extracellular concentrations can be estimated based on the mass of the brain tissue and the approximate extracellular fractional volume, under the assumption that the nanobody constructs remain in the extracellular space. As an example:

- Brain concentration in lysates 4 hours after 30 nmol/kg injection of $M1_{R56H, P96H, Y102H}$-13A7-13A7-Nb80 = 4.5 nM x 1 ml lysate = 4.5 pmol.
- Brain tissue mass ~200 mg x ~20% extracellular volume = 40 µl.
- 4.5 pmol/40 µl = 112.5 nM

If a fraction of the nanobody constructs partition into the intracellular space, the concentrations in the extracellular space would be lower. It is also possible that our capillary depletion and lysis procedure results in some loss of brain parenchymal nanobody construct, in which case we would also be underestimating the true brain extracellular concentrations. An approach like cerebral microdialysis with high molecular weight cutoff catheters [73] [74] [75] would be potentially useful for directly measuring the extracellular fluid levels and dynamics of the nanobody constructs in the living brain.

We do not know whether the histidine mutagenesis strategy employed here can be used to improve BBB transcytosis of nanobodies that bind to other receptors such as CD98hc, InsR, or IGF1R. Similarly, its generalizability to affibodies, scFvs, and other binding reagents has not been determined. There are many natural proteins such as transferrin itself with strongly pH dependent binding [5], and several other proteins have been engineered this way [34] [35] [36]. A recent report described highly cooperative pH dependent dissociation of ^{64}Cu carrying polymers in the acidic tumor microenvironment, leading to efficient PET imaging of tumor allografts in mice [76].

Full investigation of the specific cell types labeled by the nanobodies will require further work. As noted, the specificity of the 13A7 nanobody has not been fully established. Recent findings indicate that P2X7 receptors are expressed in microglia but not neurons [77]. There appears to be additional P2X7-like immunoreactivity and function in neurons [78] [79] [80], and the most parsimonious explanation for our results may be that 13A7 should be considered reflecting this sort of P2X7-like immunoreactivity. The 13A7 nanobody was primarily selected as a proof-of-concept biological macromolecule cargo with a highly abundant target in wild-type mouse brain for the purposes of assessing our TfR-binding BBB shuttle system. Thus, the specificity of the 13A7 nanobody does not affect the conclusions of the findings presented here.

The fold-increase in BBB crossing imparted by the $M1_{R56H, P96H, Y102H}$ nanobody cannot be directly calculated, but can only be estimated. As noted, we were not able to detect biotinylated $M1_{AA}$-13A7-13A7-

Nb80 (which does not bind mTfR) in capillary depleted brain lysates even after injection of 600 nmol/kg. The ELISA lower limit of quantitation was <0.166 nM, indicating <0.007% injected dose/gram of brain tissue. If passive/non-receptor-mediated BBB crossing permits BBB crossing at 0.007% injected dose/gram of brain tissue, this would indicate that 3.5% injected dose/gram of brain tissue represents 500-fold increased BBB crossing. It is difficult to compare our results indicating <0.007% injected dose/gram of brain tissue with previously reported values of 0.1 to 0.01% injected dose/gram of brain tissue for non-specific BBB crossing of biological macromolecules [1] [2]. First, prior studies typically used immunoglobulins or other proteins rather than nanobody constructs. Second, as noted above and shown in **Suppl. Fig. 2A**, capillary depletion based on quantitative Claudin 5 ELISA was ~99% complete for our studies whereas the quantitatively completeness of capillary depletion has not usually been reported for studies of this type. Third, it is not known whether 4 hours is the optimal time to measure passive/non-receptor-mediated BBB crossing. Nonetheless, based on our results, it is possible that the BBB is even tighter than previously recognized in healthy young adult mice. BBB dysfunction in aged mice and in mouse models of neurological disorders may instead be partially compromised [81]. It is not known whether partial BBB compromise is related to the reported central nervous system effects of peripherally administered macromolecules in preclinical therapeutic studies.

We noted that structures with morphologies consistent with choroid plexus appeared to be labeled by the nanobody constructs (**Suppl. Fig. 6**). Future work will be required to determine whether the nanobody constructs are undergoing blood-CSF transport as well as BBB transport.

As noted, we have not been able to directly measure on and off rate kinetics at pH 7.4 and pH 5.5 for technical reasons. Specifically, there was a baseline drift in biolayer interferometry signals at pH 5.5 that was difficult to distinguish from slower off rates. Future work will involve alternative methods for direct quantitative measurements of pH dependent on and off rates.

SUPPLEMENTAL REFERENCES

[65] D. Sehlin, X. T. Fang, L. Cato, G. Antoni, L. Lannfelt, and S. Syvänen, "Antibody-based PET imaging of amyloid beta in mouse models of Alzheimer's disease.," *Nat. Commun.*, vol. 7, p. 10759, Feb. 2016, doi: 10.1038/ncomms10759.

[66] G. Hultqvist, S. Syvänen, X. T. Fang, L. Lannfelt, and D. Sehlin, "Bivalent brain shuttle increases antibody uptake by monovalent binding to the transferrin receptor.," *Theranostics*, vol. 7, no. 2, pp. 308–318, Jan. 2017, doi: 10.7150/thno.17155.

[67] T. Logan *et al.*, "Rescue of a lysosomal storage disorder caused by Grn loss of function with a brain penetrant progranulin biologic.," *Cell*, vol. 184, no. 18, pp. 4651-4668.e25, Sep. 2021, doi: 10.1016/j.cell.2021.08.002.

[68] B. van Lengerich *et al.*, "A TREM2-activating antibody with a blood-brain barrier transport vehicle enhances microglial metabolism in Alzheimer's disease models.," *Nat. Neurosci.*, vol. 26, no. 3, pp. 416–429, Mar. 2023, doi: 10.1038/s41593-022-01240-0.

[69] A. Gehrlein *et al.*, "Targeting neuronal lysosomal dysfunction caused by β-glucocerebrosidase deficiency with an enzyme-based brain shuttle construct.," *Nat. Commun.*, vol. 14, no. 1, p. 2057, Apr. 2023, doi: 10.1038/s41467-023-37632-4.

[70] M. Pelekanos *et al.*, "Establishing sheep as an experimental species to validate ultrasound-mediated blood-brain barrier opening for potential therapeutic interventions.," *Theranostics*, vol. 8, no. 9, pp. 2583–2602, Apr. 2018, doi: 10.7150/thno.22852.

[71] S. Chen *et al.*, "A review of bioeffects induced by focused ultrasound combined with microbubbles on the neurovascular unit.," *J. Cereb. Blood Flow Metab.*, vol. 42, no. 1, pp. 3–26, Jan. 2022, doi: 10.1177/0271678X211046129.

[72] E. Porret *et al.*, "Refining the delivery and therapeutic efficacy of cetuximab using focused ultrasound in a mouse model of glioblastoma: An 89Zr-cetuximab immunoPET study.," *Eur. J. Pharm. Biopharm.*, vol. 182, pp. 141–151, Jan. 2023, doi: 10.1016/j.ejpb.2022.12.006.

[73] A. Helmy, K. L. H. Carpenter, D. K. Menon, J. D. Pickard, and P. J. A. Hutchinson, "The cytokine response to human traumatic brain injury: temporal profiles and evidence for cerebral parenchymal production.," *J. Cereb. Blood Flow Metab.*, vol. 31, no. 2, pp. 658–670, Feb. 2011, doi: 10.1038/jcbfm.2010.142.

[74] S. Magnoni *et al.*, "Tau elevations in the brain extracellular space correlate with reduced amyloid-β levels and predict adverse clinical outcomes after severe traumatic brain injury.," *Brain*, vol. 135, no. Pt 4, pp. 1268–1280, Apr. 2012, doi: 10.1093/brain/awr286.

[75] D. Cederberg, E. Visse, N. Marklund, and P. Siesjö, "Prolonged and intense neuroinflammation after severe traumatic brain injury assessed by cerebral microdialysis with 300 kDa membranes.," *J. Neuroimmunol.*, vol. 377, p. 578020, Jan. 2023, doi: 10.1016/j.jneuroim.2023.578020.

[76] G. Huang *et al.*, “PET imaging of occult tumours by temporal integration of tumour-acidosis signals from pH-sensitive 64Cu-labelled polymers.,” *Nat. Biomed. Eng.*, vol. 4, no. 3, pp. 314–324, Mar. 2020, doi: 10.1038/s41551-019-0416-1.

[77] K. Kaczmarek-Hajek *et al.*, “Re-evaluation of neuronal P2X7 expression using novel mouse models and a P2X7-specific nanobody.,” *eLife*, vol. 7, Aug. 2018, doi: 10.7554/eLife.36217.

[78] J. Sánchez-Nogueiro, P. Marín-García, and M. T. Miras-Portugal, “Characterization of a functional P2X(7)-like receptor in cerebellar granule neurons from P2X(7) knockout mice.,” *FEBS Lett.*, vol. 579, no. 17, pp. 3783–3788, Jul. 2005, doi: 10.1016/j.febslet.2005.05.073.

[79] C. M. Anderson and M. Nedergaard, “Emerging challenges of assigning P2X7 receptor function and immunoreactivity in neurons.,” *Trends Neurosci.*, vol. 29, no. 5, pp. 257–262, May 2006, doi: 10.1016/j.tins.2006.03.003.

[80] P. Illes, T. M. Khan, and P. Rubini, “Neuronal P2X7 receptors revisited: do they really exist?,” *J. Neurosci.*, vol. 37, no. 30, pp. 7049–7062, Jul. 2017, doi: 10.1523/JNEUROSCI.3103-16.2017.

[81] M. D. Sweeney, Z. Zhao, A. Montagne, A. R. Nelson, and B. V. Zlokovic, “Blood-Brain Barrier: From Physiology to Disease and Back.,” *Physiol. Rev.*, vol. 99, no. 1, pp. 21–78, Jan. 2019, doi: 10.1152/physrev.00050.2017.
